# Correlation between serum levels of C-reactive protein and neonatal pneumonia: A protocol for systematic review and meta-analysis

**DOI:** 10.1097/MD.0000000000025977

**Published:** 2021-05-21

**Authors:** Xiaowen Li, Zhong Chen

**Affiliations:** a Department of Pediatrics, West China Second University Hospital, Sichuan University; b Key Laboratory of Birth Defects and Related Diseases of Women and Children (Sichuan University), Ministry of Education, Chengdu, China.

**Keywords:** C-reactive protein, meta-analysis, neonatal pneumonia, protocol

## Abstract

**Background:**

Few studies have reported the correlation between serum levels of C-reactive protein (CRP) and neonatal pneumonia. The purpose of the present meta-analysis was to clarify whether an increased serum level of CRP accelerates the development of neonatal pneumonia.

**Methods:**

This protocol is conducted according to the preferred reporting items for systematic reviews and meta-analysis protocol (PRISMA-P) statement guidelines. Related articles were identified by searching PubMed, Embase, Cochrane Library, Web of Science, Science Direct, and CNKI databases. Two investigators extracted information according to the selection criteria and used a set of predefined criteria based on the Newcastle–Ottawa Scale (NOS) criteria to assess the studies. All calculations were carried out with Stata 12.0 (Stata Corp, College Station, TX).

**Results:**

The results of this systematic review and meta-analysis will be published in a peer-reviewed journal.

**Conclusion:**

We hypothesized that a higher serum CRP level is closely correlated with the progression of neonatal pneumonia. CRP as a general systemic inflammation biomarker may help clinicians to make difficult therapeutic decisions for neonatal pneumonia.

**Open Science Framework registration number:**

10.17605/OSF.IO/RGBMX.

## 1 Introduction

Neonatal pneumonia, which is caused by infection with viruses or bacteria, has aroused public concern due to the substantial mortality and incidence in children worldwide.^[1,2]^ The morbidity rate of neonatal pneumonia is 3.5% to 25% due to the easy complications with septicemia, respiratory distress syndrome, and others.^[3,4]^ Neonatal pneumonia is one of the main reasons leading to perinatal death. It is clinically categorized as “early onset” or “late onset”—each of which determined by factors in the intrauterine or the external environment, respectively.^[5]^ Different arrays of pathogens produce different variants of neonatal pneumonia. Early onset neonatal pneumonia is often due to pathogens aspirated by the neonate from the intrauterine environment, or from the birth canal during vaginal delivery. The risk is particularly high if the mother has chorioamnionitis—an infection of the intrauterine tissues. Late-onset pneumonia is often caused by pathogens encountered in the postnatal environment, either in the community (community-associated pneumonia), or in the hospital (hospital-associated pneumonia).

C-reactive protein (CRP), an acute phase plasma protein of the pentraxin family, is produced and released by hepatocytes and adipocytes, playing distinct roles in innate and adaptive immunity with inflammatory effects.^[6,7]^ Previous studies have identified the significant role of the serum CRP level in the development and prognosis of pneumonia, and have reported that an increased serum level of CRP may be present in patients with pneumonia.^[8,9]^ In particular, it has been found that the serum CRP level may be useful for distinguishing bacterial pneumonia from nonbacterial pneumonia in children, as higher serum CRP concentrations were observed in cases with a bacterial etiology. However, few studies have reported the correlation between serum levels of CRP and neonatal pneumonia. The purpose of the present systematic review and meta-analysis was to clarify whether an increased serum level of CRP accelerates the development of neonatal pneumonia.

## 2 Methods

This protocol is conducted according to the preferred reporting items for systematic reviews and meta-analysis protocol (PRISMA-P) statement guidelines^[10]^ and the Cochrane Handbook for Systematic Reviews of Interventions.^[11]^ Ethical approval is not required because this is a protocol for meta-analysis. The prospective registration has been approved by the Open Science Framework registries (https://osf.io/rgbmx), and the registration number is 10.17605/OSF.IO/RGBMX.

### 2.1 Selection criteria

Any randomized human-associated intervention case–control studies that involved the association of CRP serum levels with neonatal pneumonia as a primary outcome were initially taken into consideration. Studies that did not provide the number of neonatal pneumonia cases, or sufficient information about serum CRP expression levels, were not included in the meta-analysis. Extracted studies that had a considerable overlap (>50%) of study subjects or lack of complete data or unavailable data were excluded. If the same population was investigated in more than one study, only the most recent or complete study was included following careful reexamination.

### 2.2 Search strategy

Related articles were identified by searching PubMed, Embase, Cochrane Library, Web of Science, Science Direct, and CNKI databases comprehensively for all pertinent papers, which assessed the correlations between CRP serum levels and neonatal pneumonia and were published up to April, 2021. The search terms used were (“C-reactive protein”), (“infant, newborn” or “newborn infants” or “newborns” or “neonates”), and (“pneumonia” or “pneumonia, mycoplasma” or “primary atypical pneumonia” or “mycoplasma pneumonia” or “Mycoplasma pneumoniae pneumonia”) for the initial search. No limitation was set on the language of the article. Additional potentially relevant papers were further retrieved a manual search of references from the original articles.

### 2.3 Data extraction

In order to reduce bias and enhance credibility, 2 investigators extracted information according to the selection criteria separately, and arrived at a consensus on all the items through discussion and reexamination. The following relevant data were extracted from eligible studies prospectively: surname of first author, year of publication, source of publication, study type, study design, sample size, age, sex, ethnicity and country of origin, detection method of CRP serum levels, and CRP expression levels. All authors approved the final decision concerning the studies to be included.

### 2.4 Quality assessment

To decide whether a study was of high quality, the 2 authors used a set of predefined criteria based on the Newcastle–Ottawa Scale (NOS) criteria to assess the studies independently.^[12]^ The NOS criteria were scored based on 3 aspects: subject selection, 0 to 4; comparability of subject, 0 to 2; and clinical outcome, 0 to 3. Total NOS scores range from 0 (lowest) to 9 (highest). According to the NOS scores, the included studies were classified into 2 levels: low quality (0–6), and high quality (7–9). Discrepancies in the NOS scores of the included articles were resolved by an additional reviewer through discussion and consultation.

### 2.5 Statistical analysis

In order to supply quantitative evidence from all selected studies and minimize the variance of the summary, the current statistical meta-analyses was conducted utilizing a random-effects model (DerSimonian and Laird method) or a fixed-effects model (Mantel–Haenszel method) of individual study results under the situation where data from independent studies could be combined. A random-effect model was applied when heterogeneity existed among studies, while a fixed-effect model was applied when there was no statistical heterogeneity. The summary standardized mean difference (SMD) with 95% confidence intervals (CIs) was calculated for case versus control categories of serum CRP levels, with utilization of the Z-test. Subgroup meta-analyses were also conducted by ethnicity and sample size to explore potential modification effects, and heterogeneity across the enrolled studies was evaluated by the Cochran Q-statistic; *P* < .05 was regarded as statistically significant.^[13]^ As a result of the low statistical power of the Cochran Q-statistic, an *I*^2^ test was also used to reflect the possibility of heterogeneity between studies. Sensitivity analysis was performed to reflect the effect of an individual data set on the pooled SMDs. A funnel plot was constructed to assess publication bias which might affect the validity of the estimates. The symmetry of the funnel plot was further evaluated by Egger linear regression test. All tests were two-sided and a *P*-value of <.05 was regarded as statistically significant. All calculations were carried out with Stata 12.0 (Stata Corp, College Station, TX).

## 3 Discussion

The current meta-analysis considered previous findings from relevant studies in an attempt to determine the correlation between serum levels of CRP and the pathogenesis of neonatal pneumonia. CRP, a major acute phase protein, is a member of the pentraxin family and plays a central role in innate and adaptive immunity. The production of CRP is stimulated to a large extent by tumor necrosis factor-α, interleukin-6, and interleukin-1β in response to infection or inflammatory conditions.^[14,15]^ To be specific, a reduction in serum CRP levels may present a relief or alleviation of the inflammatory process, whereas persistently upregulated levels of CRP or an initial reduction followed by a further elevation might indicate persistent inflammation and poor prognosis.^[16]^ Our review process will be very rigorous and this article is a protocol of the systematic review and meta-analysis, which presents the detailed description of review implement. The results of our review will be reported strictly following the PRISMA criteria and the review will add to the existing literature by showing compelling evidence and improved guidance in clinic settings.

## Author contributions

**Data collection and original draft:** Xiaowen Li.

**Funding acquisition:** Zhong Chen.

**Study design:** Zhong Chen.

**Writing – original draft:** Xiaowen Li.
